# Supplementary material for: Effects of Different Pesticides on the Brewing of Wine Investigated by GC-MS-Based Metabolomics
Source: Metabolites. 2022 May 27;12(6):485. doi: 10.3390/metabo12060485 (PMC9228690; doi:10.3390/metabo12060485)
Supplement: Supplementary file 1 [file metabolites-12-00485-s001.zip › metabolites-1714802-supplementary.pdf]

Supporting information for: Effects of different pesticides on the brewing of wine investigated by GC-MS-based metabolomics

Table S1. Composition of the wine substances determined by GC-MS analysis.

Metabolites	VIP value	Mass	RT(min)	p value	FC(F)	FC(H)	FC(T)	FC(D)	FC(P)
acetic acid	1.35929	85	15.89187	0.001204	-	1.218966	0.593212	-	-
1,2,3-Butanetriol	1.62937	117	27.23189	6.16E-05	6.474043	∞	-	-	-
1,4-benzenediol	1.22356	478	30.1085	0.020174	2.648004	6.994054	-	1.845059	2.630955
2-Ethyl-3-hydroxypropionic acid	1.05116	72	17.9913	0.086170922	1.27479	1.686964859	-	-	-
Propanetriol	1.07307	292	18.8909	0.027363013	-	1.964843029	1.669242327	-	1.71384
2-[2-(4-Phenyl-piperidin-1-yl)-ethyl]-isoindole-1,3-dione	1.17703	86	28.0162	0.088951552	0.54649	0.411702466	0.136044877	0.639312339	0.09313
2,3-Butanediol	1.27096	117	10.04971	0.001223	1.490856	-	1.325844	-	-
2-Aminoheptanedioic acid	1.6315	84	21.62158	0.000399	-	-	-	-	1.304041
1,4-benzenediol	1.22356	478	30.1085	0.0201737	2.648	6.994054059	-	1.845058912	2.63095
2-Deoxyribose	1.41954	204	14.54985	0.000115	-	-	0.543394	0.795613	0.742132
2-Ethyl-3-hydroxypropionic acid	1.05116	72	17.99125	0.086171	1.274785	1.686965	-	-	-
2-Hydroxyglutaric acid	1.67877	85	18.5477	0.000184	-	-	0.333043	0.050698	-
2-isoindole	1.17703	86	28.01617	0.088952	0.546491	0.411702	0.136045	0.639312	0.093128
2-Isopropylmalic acid	1.1965	73	19.44863	0.008459	-	-	0.648376	-	-
2-Ketobutyric acid	1.05703	87	11.57696	0.31729	0.998579	0	0	0.401464	0.425747
2-Methyl-1,4-bis-butane	1.09476	73	19.6345	0.056	1.072938	1.284276	-	0.247777	0.207909
2-Methylbutane	1.48247	204	15.89557	0.080455	-	-	6.087121	-	2.429555
2-Mono-isobutyrin	1.27172	71	0.006078	14.51478	-	2.481711	-	0	-
2-Propanol	1.19145	52	9.413929	0.000238	∞	-	∞	-	1.079021
2-propenamide	1.02199	69	9.55486	0.13855	0.9485	-	0.684797	0.33397	0.972445
2-Thioacetyl	1.16252	131	26.23198	0.000874	4.545037	3.761568	-	3.992819	-
3,4-Dihydroxy-5-methyl-dihydrofuran	1.20534	85	16.61135	0.016374	-	-	-	0.521982	-
3-Aminoisobutyric acid	1.03666	86	17.01774	0.015792	-	-	1.080588	-	-
3-βMannobiose	1.50097	129	33.50835	0.00052	0.596111	1.184454	0.53743	1.1642	1.092885
4-Aminobutanoic acid	1.05568	174	18.67497	0.001044	1.626006	2.203817	1.761739	-	1.49575
4-Pentenoic acid	1.34571	55	13.86202	0.001182	-	-	0.642554	-	-
Acrylic acid	1.34089	305	24.20714	0.000231	-	-	-	-	38.42004
Acrylic acid	1.46438	305	24.20714	1.63E-05	-	-	9.050081	-	-
Adenine	1.16887	84	23.47097	0.060791	-	-	-	-	0.752702
Alanine	1.15297	86	11.30227	0.068218	0.965867	1.148019	0.586847	-	0.649488
Aminoacetaldehyde	1.28474	84	19.18549	0.000527	-	-	-	-	1
Arabinofuranose	1.23908	217	30.18196	0.004139	8.514555	101.0743	7.555267	2.510288	6.032124
Benzene	1.30428	105	30.43987	0.000377	1.080171	-	0.746243	-	0.986584

3-βMannobiose	1.50097	129	33.5084	0.000520304	0.59611	1.18445378	0.537430066	1.03399	1.164200438
Butanoic acid	1.16791	105	32.85359	0.032427	-	-	-	-	2.781613
Citric acid	1.5541	67	22.80685	1.84E-05	1.144088	1.285405	2.076771	1.08826	-
2-Deoxyribose	1.36672	204	14.5498	0.016070576	-	-	0.543393583	0.795612757	0.74213
D-(-)-Fructo-furanose	2.39593	217	31.89799	8.09E-05	-	-	-	0.23425	-
D-(-)-Fructose	2.20787	89	23.72675	8.17E-05	-	-	5.77366	0	-
2-Hydroxyglu-taric acid	1.08633	85	18.5477	0.025471501	-	-	0.33304287	0.050697531	-
D-(+)-Galac-turonic acid	1.53594	204	24.62484	6.87E-06	2.47611	1.635795	-	-	-
D-(+)-Trehalose	1.49968	361	33.25895	0.000497	0.168103	-	3.403952	-	-
2-Methyl-1,4-bis(trime-thylsiloxy)butane	1.09476	73	19.6345	0.055999581	1.07294	1.284275564	-	0.24777692	0.20791
D-Gluconic acid	1.49121	73	25.29977	4.8E-06	1.775537	-	-	-	-
L-lysine	1.30795	84	18.2183	0.033303379	-	-	-	-	1.60289
Disilane	1.53404	73	20.70597	0.000257	1.291529	1.303504	-	-	1.025083
D-(+)-Turanose	1.16877	135	31.16345	0.040724815	-	-	3.403952191	-	-
DL-Ornithine	1.13688	142	0.018848	22.76562	-	1.179203	0.201221	-	1.114991
D-Lyxose	1.42005	73	20.46183	0.01147	-	-	-	1	-
D-Mannitol	1.47708	319	24.40115	0.000319	1.818926	1.789018	-	-	-
N-(4-amino]bu-tyl)acetamide	1.40289	86	14.3285	0.002167231	-	-	-	-	3.21872
2-Aminoheptane-dioic acid	1.6315	84	21.6216	0.000398983	-	-	-	1.193732	1.30404
D-Xylopyranose	1.24396	73	20.5272	0.002857	1.147197	1.404651	-	-	-
D-Xylose	1.35101	204	0.003411	23.31972	-	10.18715	5.072065	-	-
Ethane	1.21401	103	24.67449	0.043297	-	-	-	0.126903	-
Ethanol	1.16516	75	15.55067	0.011141	0.746658	0.838336	0.642196	0.794435	0.649199
Ethyl βD-gluco-pyranoside	1.37168	103	28.36369	0.001349	1.310768	1.46125	-	-	1.126878
Galacto-Hexodi-aldose	1.59727	89	24.58388	8.48E-05	2.014273	1.701365	-	1.231298	-
Gln-Leu-Arg	1.47604	416	32.11	0.000209	1.313884	1.785724	-	1.168174	1.832601
Glycerol	1.18459	203	14.59304	0.009117	-	-	0.527386	0.721748	0.682495
Glyceryl-glyco-side	1.57621	103	28.53803	0.000513	1.592898	1.808451	-	-	1.069888
Glycolic acid	1.80691	87	2.45E-07	11.5817	-	∞	7.438813	-	-
Hydroxylamine	1.07344	119	11.65536	0.12117	-	-	-	-	0.386774
L-5-Oxoproline	1.6533	156	2.06E-05	18.63411	-	1.550549	-	-	-
Lactic Acid	1.29894	87	10.53345	0.011053	1.053966	-	0.536054	-	0.777306
L-Aspartic acid	1.42818	73	18.47547	1.55E-05	1.578264	1.41351	-	0.719657	-
L-Glutamic acid	1.32539	128	19.91577	0.001841	1.402976	1.571477	-	-	1.252725
L-Lysine	1.40933	128	24.06302	0.000697	-	-	0.437525	-	-
L-Norvaline	1.23768	114	13.39086	0.004595	1.080971	1.189563	0.597305	-	0.638046
L-Ornithine	1.11485	70	19.85138	0.004059	-	-	2.36951	1.599107	2.997885
L-Phenylalanine	1.3668	91	20.16594	0.000141	1.338407	1.56091	-	0	0
L-Proline	1.1689	143	15.00047	0.019086	-	-	0.437599	-	1.410908
L-Sorbopyranose	1.62078	73	32.57183	0.004587	-	-	0.222118	0.49997	-
Malic acid	1.04278	73	17.97557	0.017644	0.302842	-	0.675167	1.046246	0.800688
meso-Erythritol	1.44533	217	0.000907	18.32941	-	1.50405	1.174095	0.580686	-
Methadone	1.15545	105	31.86186	0.073499	-	-	-	-	0.318151
Methoxyacetic acid	1.37863	89	21.38958	0.069914	-	-	-	0.495796	-

Methyl xylopyra-noside	1.2358	204	32.34565	0.005389	1.88299	2.196754	-	1.583434	-
Muscimol	1.14854	73	17.34786	0.036742	5.008348	∞	7.387087	-	-
N-Formylglycine	1.55784	73	20.15992	2.36E-05	-	-	0	-	-
Oxalic acid	1.11056	73	26.35881	0.009028	1.351243	-	-	1.230938	-
Pentanedioic acid	1.25701	84	19.26835	0.000903	-	-	0.662815	-	-
Pentasiloxane	1.60646	73	12.59265	4.92E-05	1.911732	1.975021	-	1.754133	1.569799
Pregna-3,5-dien-20βol	1.54027	117	27.28645	0.05605	-	-	-	1.921015	-
Propanoic acid	1.28281	73	28.82706	1.52E-06	7.027658	5.25342	0.433753	3.664049	0.501449
Pyruvaldehyde	1.42576	100	14.38859	0.001168	-	-	0.39324	0.77558	0.692534
Silane	1.1442	73	0.018714	26.63692	-	1.520408	-	1.37774	-
S-Methyl-L-cys-teine	1.06995	211	13.54728	0.028567	0.378942	-	-	-	-
βD-(+)-Mannopy-ranose	1.45422	204	24.79585	7.45E-05	-	-	3.332321	-	-
βD-(+)-Talopyra-nose	1.37416	73	23.96887	0.000116	1.041925	-	0.363394	-	-
βD-(+)-Xylopyra-nose	1.49445	149	23.31341	0.000969	1.311775	0.25001	0	0.52295	-
βD-Glucopyra-nose	1.39905	204	23.85569	0.000815	-	-	2.509828	-	-
βL-Idopyranu-ronic acid	1.25716	73	28.65005	0.002731	-	-	0.517919	-	-
βN-Formyl-L-ly-sine	1.20366	84	18.21829	0.009274	-	-	1.97199	-	-

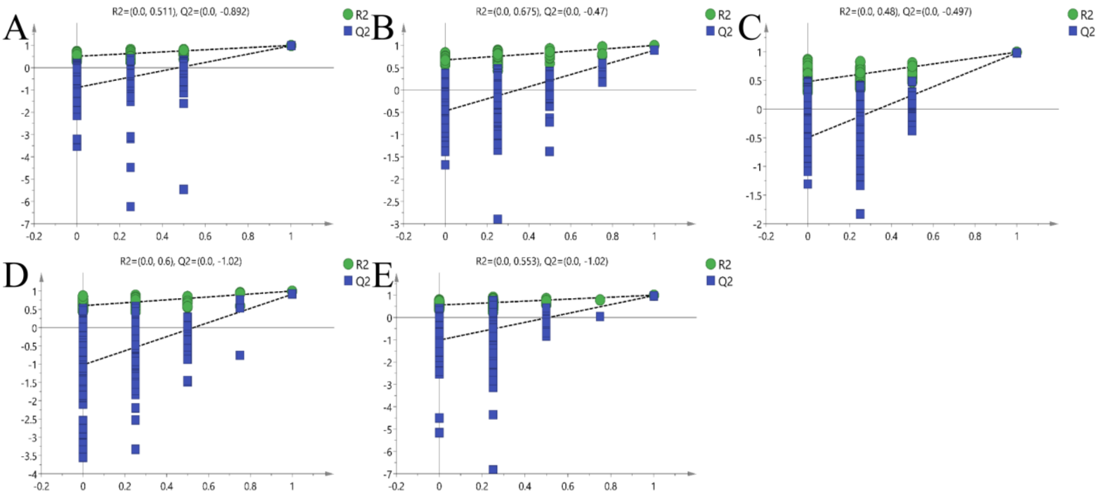

Figure S1. Response permutation testing (RPT). RPT is a random ranking method used to evaluate the accuracy of (O)PLS models and avoid supervised learning methods, thus obtaining not accidental classification. (**A**) CK and F; (**B**) CK and H; (**C**) CK and T; (**D**) CK and D; (**E**) CK and P. Note: CK, blank control group; F, flutriafol; H, hexaconazole; T, tebuconazole; D, difenoconazole; P, propiconazole.
